# Ecological Momentary Assessment of Momentary Associations Between Availability of Physical Activity Space and Physical Activity Opportunities Among Children from Rural, Urban, and Suburban Locales

**DOI:** 10.3390/ijerph21121586

**Published:** 2024-11-28

**Authors:** Ann Kuhn, Yan Wang, Rachel Deitch, Amy Zemanick, Genevieve Dunton, Lindsey Turner, Erin R. Hager

**Affiliations:** 1 Department of Pediatrics, University of Maryland School of Medicine, Baltimore, MD 21201, USA; yanwang20@email.gwu.edu (Y.W.); rdeitch1@jhu.edu (R.D.); ehager1@jhu.edu (E.R.H.); 2 Department of Exercise and Nutrition Sciences, University at Buffalo, The State University of New York, Buffalo, NY 14214, USA; 3 Department of Prevention and Community Health, Milken Institute School of Public Health, George Washington University, Washington, DC 20052, USA; 4 Department of Population, Family and Reproductive Health, Johns Hopkins Bloomberg School of Public Health, Baltimore, MD 21205, USA; 5 Department of Epidemiology, Johns Hopkins Bloomberg School of Public Health, Baltimore, MD 21205, USA; 6 Department of Population and Public Health Sciences, Keck School of Medicine, University of Southern California, Los Angeles, CA 90033, USA; dunton@usc.edu; 7 College of Education, Boise State University, Boise, ID 83725, USA; lindseyturner1@boisestate.edu

**Keywords:** physical activity, ecological momentary assessment, geographic locale, children, physical activity accessibility

## Abstract

Using Ecological Momentary Assessment (EMA), this study examined associations between momentary availability of physical activity (PA) space and accessibility of PA opportunities among 608 elementary and middle school students who were participating in an obesity prevention trial in one mid-Atlantic state in the U.S. Smartphones prompted EMA surveys at random times to assess children’s perceived availability of PA space and accessibility of PA opportunities during out-of-school time, three to seven times each day over seven days. Multilevel logistic regression, which accounted for multiple responses per student, examined within- and between-person relations as well as the moderating effects of locale. The participants (*M* age = 10.88 years) lived in suburban (64%), rural (23%), and urban locales (13%). PA space availability was associated with greater PA opportunity accessibility (within-person OR = 9.82, *p* < 0.001; between-person OR = 22.61, *p* < 0.001). Locale moderated within-person relationships (*p* < 0.001), indicating that urban students with space were unable to use it or could be active but were without space. These findings advance our knowledge of temporal and environmental aspects related to childhood PA across diverse locales and can be used by policymakers to make informed decisions to ensure the use of age-appropriate, high quality, and safe spaces, particularly for children in urban areas.

## 1. Introduction

Prior to the start of the COVID-19 pandemic, nearly 76% of U.S. children and adolescents were not meeting national recommendations to engage in 60 min of moderate to vigorous physical activity (PA) per day [1,2]. In recent years, as a result of COVID-19 pandemic control policies, U.S. children have become more sedentary and are spending less time engaging in PA [3,4]. Physical inactivity can increase the risk of obesity, cardiovascular disease, type 2 diabetes, many types of cancers, and have detrimental impacts on mental and emotional health in children [1]. This study uses a socio-ecological approach to conceptualize influences on the PA of children [5] as the built environment is a key determinant of PA [6], including the geographic locale in which an individual lives (i.e., rural, suburban, and urban locales) [7]. However, research comparing PA levels between rural and urban children is inconsistent [8,9]. For example, one study using accelerometer data found that moderate to vigorous PA (MVPA) was higher among urban adolescents when compared to those living in non-urban areas [8], whereas other work has shown higher MVPA among rural students when compared to those living in urban areas [9]. These inconsistent findings regarding differences in PA by locale could be due to differences in how individual children interact with their built environment.

Environmental factors linking geographic locale and PA levels in children include both availability of space and accessibility to PA opportunities [10,11,12]. Availability of space is often operationalized as the presence of space for PA [13], while accessibility is operationalized as the ease with which PA opportunities can be reached in a given space [14]. Overall, studies that examined the built environment and geographic locale have suggested that associations between elements of the built environment (e.g., the availability of space and accessibility to PA opportunities) and PA differ between rural, urban, and suburban locales [15,16,17]. Moreover, rural–urban differences in the associations between the built environment and opportunities to be active are complex and can sometimes show opposite correlational trends (e.g., street connectivity and active commuting) [18]. For instance, walking or cycling in rural locales may not be limited by availability of space, but rather due to traffic safety or a lack of bike lanes, which limits accessibility [15]. Likewise, children and adolescents living in rural locales may have an availability of spaces to be active on school grounds but may lack transportation or live too far from the school for these spaces to be accessible during out-of-school hours [12]. In urban locales, children report a limited availability of space for PA, or if they have space (e.g., a neighborhood park), safety concerns may inhibit their usage (i.e., low accessibility) [19]. Across locales, access to PA opportunities may also be restricted by school authority or parent rules that prohibit indoor play [20,21]. Therefore, availability and accessibility to space for PA may have different associations with PA among children who live in rural, urban, and suburban locales, supporting the need for more research in this area. To date, these constructs have primarily been evaluated with respect to the community or neighborhood environment, including green space or park availability (is it nearby?) or playground or fitness center accessibility (is it open?), with limited focus on availability and accessibility in the home environment, warranting the need for the current study.

One criticism of previous studies that examine environmental correlates of PA is that they have measured behaviors and contexts at a single time point or very infrequently [22]. This leads to aggregated estimates of an individual’s average levels of PA behaviors at a single time point, which may conceal temporal and spatial variations specific to the individual. It is increasingly recommended that contexts are measured on a momentary basis to capture time-varying and within-person processes underlying emotional, cognitive, or behavioral experiences occurring in those contexts [22,23]. To address these criticisms, Ecological Momentary Assessment (EMA) has emerged as a popular strategy for capturing repeated self-reports of real-time information on contextual factors or behaviors [24], such as physical activity in children [25,26]. EMA is characterized by four key features: (1) data collection in natural environments; (2) assessments that focus on the individual’s current state, experience, or behavior; (3) assessments that are either event-based, time-based, or randomly prompted; and (4) multiple, repeated assessments that occur over time [27]. EMA has been used to examine the social and built environmental factors associated with PA among adults [28], as well as to describe the physical contexts of children’s self-reported PA (i.e., at school, at home, and outdoors) [29], but it has not been used to examine children’s PA space availability and accessibility to PA opportunity across diverse locales. EMA’s methods offer the opportunity to examine the extent to which availability of space for PA and accessibility to PA opportunities co-occur in time for children in rural, urban, and suburban locales.

Given that environmental factors influencing PA may vary by geographic characteristics, it is important to investigate whether the association between the availability of space for PA and the accessibility of PA opportunities varies by urban vs. suburban vs. rural locale. Furthermore, there is a need to address methodological weaknesses of previous research on environmental factors related to children’s PA by utilizing momentary measures to understand the temporality of contextual factors related to PA. Better discernment of these associations could inform future context-specific PA interventions for children in rural, urban, and suburban locales. Therefore, the purpose of this study was to examine momentary associations between availability of space for PA and accessibility of PA opportunities by locale (rural, suburban, and urban locales). It was hypothesized that among individual children (within-persons), during moments where space for PA is available, there will be higher reported accessibility of PA opportunities. It was also hypothesized that, on average (between-persons), children with greater availability of space for PA will also have greater accessibility to PA opportunities. Furthermore, it was hypothesized that locale would moderate the within- and between-person associations between availability of space and accessibility to PA opportunities. Limited research has been done in suburban locales. However, based on prior research findings that children and adolescents living in rural locales may have a greater availability of PA space, but limited accessibility to PA opportunity, whereas those in urban locales may have limited PA space and limited accessibility of PA opportunity, it was hypothesized that positive associations between accessibility of PA opportunity and the availability of space would be stronger in suburban locales compared to rural and urban locales.

## 2. Materials and Methods

### 2.1. Participants

The current analyses used data collected from students aged 9–15 years who participated in the Wellness Champions for Change (WCC) study [30]. WCC was a school-based cluster randomized controlled trial examining strategies for enhancing the implementation of local wellness policies in schools by training teachers and students as champions who lead wellness teams [30]. EMA measures were employed to test a secondary hypothesis that health-promoting environments would moderate the impact of a school-based intervention. Participants were from 33 elementary and middle schools from five geographically-diverse school districts in a Mid-Atlantic state. A total of 964 students were recruited and completed baseline assessments (i.e., completed surveys, wore an accelerometer, and completed EMA prompts using a mobile phone) in either the spring or fall of 2017, 2018, and 2019 through in-person recruitment in classrooms, during lunch, and at after-school events for families or through school announcements, posters, flyers, and informational pamphlets. Interested students provided parental contact information then the study was discussed with the parents/guardians. Written informed consent and assent was provided by the parent/guardians and students, respectively. Following the baseline evaluation, schools underwent randomization. By design, a subset of students (n = 760, 78.8%) was randomly selected from the original sample to participate in the EMA portion of baseline data collection. Approval was granted by the institutional review board of the university (ethics approval number: HP-00067626) and five participating school districts. Participants were provided a $15 gift card for completing the baseline survey and another $15 gift card for wearing the accelerometer. There were no incentives for completing the EMA portion of the study.

### 2.2. Data Collection Procedures

Students were provided an inexpensive Android smartphone with the MovisensXS App (Karlsruh, Germany) that prompted EMA surveys randomly during out-of-school time across seven days in total; prompts randomly occurred seven times on weekend days (between 9 am and 8 pm) and three times on school days (between 3 pm and 8 pm), an acceptable number for children [31,32]. After the auditory prompt, students were instructed to complete a short EMA survey on the smartphone app. The survey included questions about current PA behaviors and availability/accessibility of environments. When prompted via smartphone, students could delay answering up to 9 min and would be re-prompted one time per prompt. The student response rate was calculated based on the number of EMA prompts answered out of the total possible prompts sent over the study period.

### 2.3. Measures

#### 2.3.1. Availability of Space for Activity and Accessibility of PA Opportunity

The questions used in the current analyses were: (1) Availability of space (the ordinal variable), with the question: “How much space is there to run around and be active right now?” and three response options: no space, a little space, or a lot of space, and (2) Accessibility to PA opportunities (binary variable), with the question: “If you wanted to run around and be active right now, could you?” and response options of yes or no. These questions were reviewed with a focus group of 10 3rd, 6th, and 9th grade students for comprehension and then pilot-tested with a larger cohort of 58 3rd, 6th, and 9th grade students in conjunction with other measures developed for this study [33]. Using the pilot data, response options were checked for completeness and the distribution of responses.

For analyses, availability of space was collapsed into a binary variable and categorized as 1 = “a little or a lot of space” and 0 = “no space”. Accessibility to PA opportunities was coded as 1 = “yes” and 0 = “no”.

#### 2.3.2. Student Demographics and Anthropometric Measures

Student-level demographic data were collected via electronic survey and included: age, gender, and race. Students’ height (cm) and weight (kg) were measured in triplicate by a trained research assistant using a portable stadiometer (Shorr Productions, Olney, MD, USA) and a standard scale (300 GS, Tanita Corp, Tokyo Japan). These measurements were used to calculate students’ BMI-for-age percentiles, which categorized students as underweight, healthy weight, overweight, or obese, based on CDC cutoffs [34]. Participants’ locale was determined by geocoding participants’ home address using ArcGIS software by ESRI (Redlands, CA, USA). Geocoding is the process of converting tabular geographic information into projected geographic data [35]. Address information listed in columns and rows was matched to open-source reference data through Maryland’s Geocoder, then projected to the map as points. The location of each point was referenced to locale boundaries obtained from the National Center for Education Statistics (NCES) [36] to determine if the address is rural, urban, or suburban. All geographic data processing and cartography was performed with Esri’s ArcGIS Pro.

#### 2.3.3. School Demographics

School-level demographic data, including eligibility for free and reduced-price meal services (FARMS), as a proxy for school-level poverty were collected from the National Center for Education Statistics (NCES) or school websites. FARMS was recoded into three categories: (1) lower poverty: 0–39.9%, (2) moderate poverty: 40–74.9%, and (3) higher poverty: 75% or more. If home locale was not able to be geocoded, the locale of the student’s school address, as determined by the NCES website [36], was used in the analysis.

### 2.4. Statistical Analysis

Descriptive analyses were conducted first. Chi-square tests for categorical variables and t-tests for continuous variables assessed differences in demographic variables between the participants included and excluded from the analysis. Multilevel logistic regression models were used to assess whether missing and non-missing data differed by demographic variables (i.e., gender, race, school type, weight status, % FARMS) or prompt time (i.e., time of week and time of day).

To account for repeated measures of the EMA prompts, multilevel logistic regression modeling was performed since the dependent variable (accessibility of PA opportunity) was a binary variable. In addition, person-mean centering of the time-varying predictor (availability of space for activity), also a binary variable, was conducted to find the average response for each participant and the variability of their responses within each participant [37]. To do so, a mean of each predictor was created for each participant (the average of all responses for a single construct) and this measured the deviation of each participant’s specific response at each assessment from the person-level average. Both the average response (between-person effect) and the deviation of the response from mean (within-person effect) were included in the multi-level modeling in relation to the outcome (accessibility of PA opportunity). Within-person effects represent—at any given prompt—whether having a greater proportion of availability of space for PA, compared to their own average, is associated with a higher likelihood of having accessible PA. Between-person effects represent whether the participants who had a higher proportion of available of space for PAs had a higher likelihood of accessible PA at any given prompt.

To examine the moderating effect of locale, a series of models were calculated. Model 1 included only availability of space for PA as a predictor; Model 2 included both the availability of space for PA and locale (a three-category variable including rural, suburban, and urban locales) as predictors. Likelihood Ratio Tests (LRT) assessed the overall significance in relation to locale and accessibility of PA opportunity and Wald tests assessed pairwise differences between locale categories. Model 3 was the same as Model 2 (with availability of space for PA and locale), plus interaction terms between the availability of space for PA and locale for within-person and between-person relations. Similarly, LRT assessed the overall significance in the interactions and Wald tests assessed pairwise comparison between locale categories. Significant interactions would be followed by stratified analyses by locale as post-hoc analyses. Covariates were chosen based on whether missing and non-missing data differed by demographic variables and prompt time and were included in all models. Statistical analyses were conducted using software IBM SPSS version 26.

## 3. Results

### 3.1. Sample Characteristics

**Figure 1:**
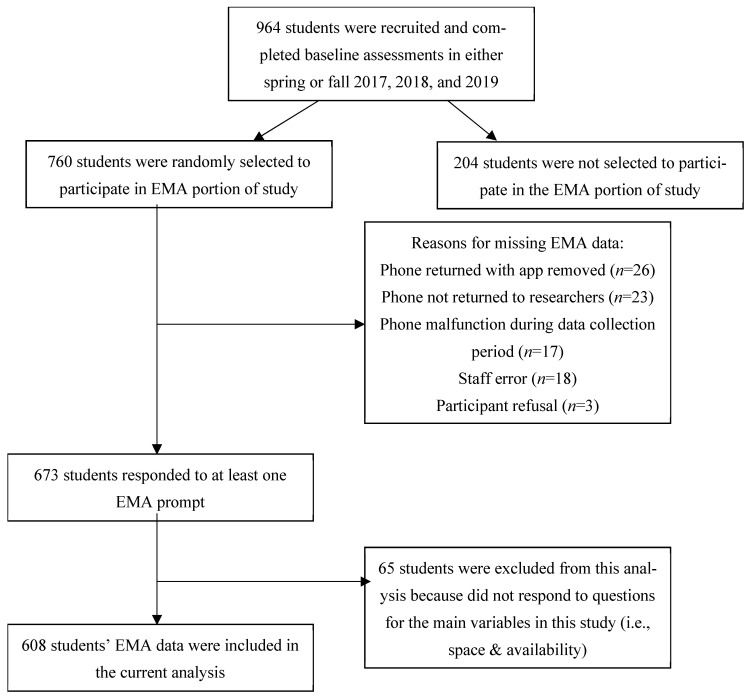
Participant flow chart.

Of the 760 students who were randomized to receive a phone, 673 responded to at least one EMA prompt (participant flow chart is shown in Figure 1). Reasons for missing data (or not having data for at least one EMA prompt) include: phone returned with app removed (unclear if student deleted app or it was not properly loaded, n = 26), phone not returned to researchers (n = 23), phone malfunction during the data collection period (n = 17), staff error (e.g., same ID assigned to more than one participant, errors downloading data; n = 18), and participant refusal (n = 3). Of the 673 students who responded to at least one EMA prompt, 65 were excluded since they did not respond to any questions for the main variables in this analysis (i.e., space and accessibility), resulting in a total of 608 participants in the analytic sample (inclusion rate: 608/760 = 80%). This sample is described in Table 1. The mean age was 10.88 years (SD = 1.54) and a majority of the students were female (59%) and had a healthy weight (53%). The race/ethnicity of the sample included 29.6% non-Hispanic Black, 27.1% non-Hispanic more than one race/ethnicity, 20.7% non-Hispanic White, 16.8% Hispanic/Latino, 1.0% non-Hispanic Asian, 0.3% non-Hispanic Middle Eastern or North American, 0.2% non-Hispanic Native American/Alaskan native, 3.1% did not know their race/ethnicity, and 1.2% race/ethnicity was not collected. For analyses, race was categorized as “white” and “non-white”, and 79.3% were of a non-white race/ethnicity. In terms of the characteristics of schools attended, 60% of students attended an elementary school, and 61% were from a school with moderate to high poverty levels (FARMS rate of 40% or more). Most students lived in suburban locales (64%), while the remaining were located in rural locales (23%) or urban locales (13%). Fifty-nine students were missing geocoded home addresses, so a locale based on the school’s address was used instead. Chi-square and independent t-tests showed that included participants (n = 608) were more likely to be in elementary schools compared to excluded participants (n = 152, *p* = 0.015); however, no other significant differences were found between included and excluded participants based on gender, locale, race/ethnicity, and weight status, nor by school-level poverty (FARMS). Given the noted differences, school level was added to the models as a covariate.

### 3.2. Ecological Momentary Assessment (EMA)

Among the 673 participants with EMA data, 12,802 individual EMA responses were recorded. Individual response rates for each participant were calculated by dividing the number of prompts that individuals responded to by the number that they received. All individual response rates were averaged to calculate the overall EMA response rate, which was 41% (SD = 26%). Multilevel logistic regression models indicated that participants had greater odds of missing prompts on weekdays (OR 0.88 [CI: 0.81–0.95], *p* = 0.002) and if they attended a higher poverty school (OR 0.67 [CI: 0.53–0.85], *p* < 0.001). Participants had greater odds of missing prompts in the afternoons compared to mornings or evenings (OR 1.22 [CI: 1.13–1.32], *p* < 0.001). Therefore, school poverty, time of week, and time of day were added to the models as covariates. The final analytic sample of 608 included 5051 individual EMA responses, after removing 65 participants who did not respond to a single EMA response (which accounted for 936 individual lines of EMA data) and an additional 6815 lines of EMA data from included participants because they were missing responses to the availability and accessibility items (See Table 2). Of the 5051 individual EMA responses included in the analysis, 2789 were completed on the weekend and 2262 were completed on weekdays. Furthermore, of the prompts completed on weekends, 869 prompts were completed in the morning, while 1920 prompts were completed during afternoon/evening. On weekdays, all 2262 prompts were completed in the afternoon and evenings.

### 3.3. Availiability of Space and Acccessibility of PA Opportunities (Within- and Between-Person)

In Model 1 (Table 3), during times when children had more space available for PA than average, they had nine times greater odds of having accessible PA opportunities (within-person OR = 9.82, *p* < 0.001). Also, among children who, on average compared to others, had more space available for PA, they had 22 times greater odds of having accessible PA opportunities (between-person OR = 22.61, *p* < 0.001).

In model 2 (Table 3) when locale was added to the model, a Likelihood Ratio Test (LRT) showed that there was an overall significant relation between locale and accessibility of PA opportunities (-2LL = 20.61, df = 2, *p* < 0.001). Children living in an urban locale had two times greater odds of having accessibility of PA opportunities compared to children in suburban locales (OR = 2.09, *p* = 0.007). However, there was no significant difference in availability of PA comparing rural locale to suburban locale (*p* > 0.05).

### 3.4. Moderating Effect of Locale

In Model 3 (Table 4), the moderating effect of locale was assessed. There was a significant cross-level interaction for within-person availability of space for PA and locale (-2LL = 39.959, df = 7, *p* < 0.001), but no significant interaction for between-person availability of space and locale. To better understand the nature of the interactions, models were stratified by geographic locale (Table 5). There was a significant relation between within-person availability of space and accessibility of PA opportunity in urban (OR = 3.50, *p* < 0.001; 3.5 times greater odds of having accessibility compared to rural or suburban), rural (OR = 12.07, *p* < 0.001; 12 times greater odds of having accessibility compared to urban and suburban) and suburban locales (OR = 11.76, *p* < 0.001; 11 times greater odds of having accessibility compared to rural and urban), separately. However, the relation was about 70% times weaker in urban locales compared to suburban locales (OR = 0.33, *p* < 0.001, Table 4); there was no significant difference between suburban locale and rural locale in the strength (OR = 0.92, *p* = 0.781, Table 4).

## 4. Discussion

Using EMA to assess the availability of space for PA and accessibility to PA opportunities, this study yielded both within-person (momentary-level) and between-person (person-level) associations. The study’s findings suggest that availability of space for PA is associated with children’s accessibility to PA opportunities. Furthermore, locale moderated the relationship between availability of space and accessibility to PA opportunities, such that children in urban locales reported having space, but not being able to access it for PA.

This study found that children generally reported being able to access space for PA if it was available. These findings are in line with previous studies that have shown the importance of having space available for PA [38,39], as perceptions of having space available have been found to be associated with a greater likelihood of accessing it for PA opportunities. Each locale is known to have unique characteristics that promote PA; for example, urban locales may promote more active transportation [40], whereas rural locales may promote less screen time [41]. Thus, researchers should consider capitalizing on enhancing characteristics that are unique to each locale to inform PA interventions specific to rural, urban, and suburban locales.

This study also found that locale moderated the within-person (momentary) association between availability of space for PA and accessibility of PA opportunity, such that when students in urban locales had space, they were less likely to access it for PA opportunities or might not have been able to be active compared to the children in suburban or rural locales. This finding is in line with our hypothesis, which stated that the associations would be stronger in suburban locales compared to urban locales, as well as previous research showing that the suburban built environment was the most conducive to promoting childhood PA [42]. Urban locales are more likely to be perceived as unsafe, compared to suburban locales [19,43], which could explain why students reported having space, but were not able to access it for PA opportunities. Furthermore, students could have also had parental restrictions that did not allow them to be active indoors (e.g., running around in the house or during a church service) [21]. Future research should examine specific factors that may pose as a barrier to accessing space for PA opportunities, and specifically target urban locales within which to intervene.

This study has several limitations. First, the mean EMA response rate is low compared to response rates in other EMA studies that used mobile devices to prompt participants (range: 69% to 84%) [44]. However, this rate is an estimate using the maximum possible prompts allowed for each participant as the denominator and it is unknown whether non-responses were due to participant non-response or device-related issues (e.g., the phone was turned off or not charged). Furthermore, participants were not provided incentives that were based on the number of prompts that were completed. Perhaps providing a better incentive system would increase the EMA response rate. Additionally, the EMA questions were brief and did not include detailed contextual information. Future work should examine accelerometer-derived PA in association with availability of space and accessibility to PA opportunities to understand more about momentary associations between availability, accessibility, and children’s objectively measured PA by locale.

## 5. Conclusions

This study is unique in its use of momentary measures, which allowed for the examination of the temporal link between PA space availability and accessibility to PA opportunities. These findings advance our knowledge of the environmental aspects related to PA across diverse locales and can be used by policymakers to make informed decisions about the use of space in their communities. Researchers can also use these findings to inform new childhood PA interventions specific to rural, urban, and suburban locales. In developing new childhood PA interventions, special consideration should be given to rural–urban–suburban differences in the built environment. In particular, children in urban environments may need additional environmental supports that provide age-appropriate, high quality, safe spaces for physical activity.

## Figures and Tables

**Table 1:** Description of Student Sample (n = 608).

	Mean (Range) or N (%)
Age (years)	10.88 (9–15)
Gender	
Male	252 (41%)
Female	356 (59%)
Race/ethnicity	
Non-white	182 (79%)
White	126 (21%)
Weight status	
Underweight	4 (1%)
Healthy weight	248 (53%)
Overweight	83 (18%)
Obese	131 (28%)
Locale	
Rural locale	155 (23%)
Urban locale	90 (13%)
Suburban locale	428 (64%)
Characteristics of Schools Attended by Student Sample	
School type	
Elementary	364 (60%)
Middle	244 (40%)
FARMS %	
Lower poverty (0–39%)	236 (39%)
Moderate poverty (40–74.9%)	188 (30%)
Higher poverty (75% or more)	184 (31%)

*Note.* FARMS % = Percent of student population that qualified for free and reduced meal services.

**Table 2:** Smartphone Responses (*n* = 5051).

Factor	Question	Response Options	Most Frequent Response
Availability of space	How much space is there to run around and be active right now?	No space A little space A lot of space	40% (A lot of space) 40% (A little space)
Accessibility of PA opportunity	If you wanted to run around and be active right now, could you?	4. No 5. Yes	70% (Yes)

*Note.* PA = physical activity.

**Table 3:** Models 1 & 2: Multilevel binary logistic regression models showing within- and between-person relationships between space, accessibility, and locale.

	OR (95% CI)	*p*
Model 1: Availability of space in relation to accessibility of PA opportunity		
Within-person effects		
WP Availability of space	**9.82** **(7.87–12.25)**	<0.001 **
Between-person effects		
BP Availability of space	**22.61** **(12.99–39.35)**	<0.001 **
Model 2: Availability of space and locale in relation to accessibility of PA opportunity		
Within-person effects		
WP Availability of space	**9.89** **(7.92–12.34)**	<0.001 **
Between-person effects		
BP Availability of space	**23.17** **(13.31–40.31)**	<0.001 **
BP Suburban locale (ref)	-	-
BP Urban locale	**2.09** **(1.23–3.57)**	**0.007**
BP Rural locale	0.92 (0.61–1.37)	0.674

*Note.* ** *p* < 0.01. Significant regression coefficients are in bold fonts. Covariates = school type, & FARMS, time of week, and time of day. PA = physical activity; WP = within persons; BP = between persons. When suburban locale was used as the reference in model 2, a significant association for living in an urban locale was found (OR = 2.09 [1.23–3.57], *p* = 0.007).

**Table 4:** Model 3: Multilevel binary logistic regression model showing a cross-level interaction for within-person availability of space for PA and locale.

	OR (95% CI)	*p*
Between-person effects		
BP Availability of space	**59.88** **(15.68–228.61)**	<0.001 **
BP Locale		
BP Suburban (ref)	-	-
BP Urban	1.85 (0.95–3.63)	0.072
BP Rural	0.93 (0.62–1.39)	0.713
BP Space x locale interaction terms		
BP Space x suburban locale (ref)	-	-
BP Space x urban locale	0.17 (0.02–1.22)	0.078
BP Space x rural locale	0.37 (0.08–1.67)	0.196
Within-person effects		
WP Availability of space	**12.35** **(7.43–20.49)**	<0.001 **
WP Space x locale interaction terms		
WP Space x suburban locale (ref)	-	-
WP Space x urban locale	**0.33** **(0.14–0.65)**	<0.001 **
WP Space x rural locale	0.92 (0.52–1.64)	0.781

*Note.* ** *p* < 0.01. Significant regression coefficients are in bold fonts. Covariates = school type, & FARMS, time of week, and time of day. PA = physical activity; WP = within persons; BP = between persons. When using suburban locale as the reference, a significant cross-level interaction for within-persons space for PA and living in an urban locale (OR = 0.33, *p* < 0.001) was found.

**Table 5:** Models 4a–c: Multilevel binary logistic regression models stratified by locale showing significant relations between within-person availability of space and accessibility of PA opportunity in urban, rural, and suburban locales.

	OR (95% CI)	*p*
Model 4a: Rural locale		
BP Availability of space	**55.83** **(15.75–197.88)**	<0.001 **
WP Availability of space	**12.07** **(7.31–19.92)**	<0.001 **
Model 4b: Urban locale		
BP Availability of space	**8.84** **(2.55–30.66)**	<0.001 **
WP Availability of space	**3.50** **(1.98–6.18)**	<0.001 **
Model 4c: Suburban locale		
BP Availability of space	**23.44** **(11.50–47.80)**	<0.001 **
WP Availability of space	**11.76** **(8.91–15.54)**	<0.001 **

*Note.* ** *p* < 0.01. Significant regression coefficients are in bold fonts. Covariates = school type, & FARMS, time of week, and time of day. PA = physical activity; WP = within persons; BP = between persons.

## Data Availability

The datasets used and/or analyzed during the current study are available from the corresponding author on reasonable request.
